# Gene expression profiles define molecular subtypes of prostate cancer bone metastases with different outcomes and morphology traceable back to the primary tumor

**DOI:** 10.1002/1878-0261.12526

**Published:** 2019-06-27

**Authors:** Elin Thysell, Linda Vidman, Erik Bovinder Ylitalo, Emma Jernberg, Sead Crnalic, Diego Iglesias‐Gato, Amilcar Flores‐Morales, Pär Stattin, Lars Egevad, Anders Widmark, Patrik Rydén, Anders Bergh, Pernilla Wikström

**Affiliations:** ^1^ Department of Medical Biosciences Pathology Umeå University Sweden; ^2^ Department of Mathematics and Mathematical Statistics Umeå University Sweden; ^3^ Department of Surgical and Perioperative Sciences Orthopaedics Umeå University Sweden; ^4^ Department of Drug Design and Pharmacology Faculty of Health and Medical Sciences University of Copenhagen Denmark; ^5^ Department of Surgical Sciences Uppsala University Sweden; ^6^ Department of Pathology and Cytology Karolinska University Hospital Stockholm Sweden; ^7^ Department of Radiation Sciences, Oncology Umeå University Sweden

**Keywords:** bone metastasis, gene expression, gene set enrichment analysis, morphology, survival, unsupervised cluster analysis

## Abstract

Bone metastasis is the lethal end‐stage of prostate cancer (PC), but the biology of bone metastases is poorly understood. The overall aim of this study was therefore to explore molecular variability in PC bone metastases of potential importance for therapy. Specifically, genome‐wide expression profiles of bone metastases from untreated patients (*n = *12) and patients treated with androgen‐deprivation therapy (ADT, *n = *60) were analyzed in relation to patient outcome and to morphological characteristics in metastases and paired primary tumors. Principal component analysis and unsupervised classification were used to identify sample clusters based on mRNA profiles. Clusters were characterized by gene set enrichment analysis and related to histological and clinical parameters using univariate and multivariate statistics. Selected proteins were analyzed by immunohistochemistry in metastases and matched primary tumors (*n = *52) and in transurethral resected prostate (TUR‐P) tissue of a separate cohort (*n = *59). Three molecular subtypes of bone metastases (MetA‐C) characterized by differences in gene expression pattern, morphology, and clinical behavior were identified. MetA (71% of the cases) showed increased expression of androgen receptor‐regulated genes, including prostate‐specific antigen (PSA), and glandular structures indicating a luminal cell phenotype. MetB (17%) showed expression profiles related to cell cycle activity and DNA damage, and a pronounced cellular atypia. MetC (12%) exhibited enriched stroma–epithelial cell interactions. MetB patients had the lowest serum PSA levels and the poorest prognosis after ADT. Combined analysis of PSA and Ki67 immunoreactivity (proliferation) in bone metastases, paired primary tumors, and TUR‐P samples was able to differentiate MetA‐like (high PSA, low Ki67) from MetB‐like (low PSA, high Ki67) tumors and demonstrate their different prognosis. In conclusion, bone metastases from PC patients are separated based on gene expression profiles into molecular subtypes with different morphology, biology, and clinical outcome. These findings deserve further exploration with the purpose of improving treatment of metastatic PC.

**Abbreviations**
ADT: androgen‐deprivation therapy
AR: androgen receptor
CRPC: castration‐resistant prostate cancer
GSEA: gene set enrichment analysis
GS: Gleason score
MetA-C: bone metastasis subtypes A-C
OPLS‐DA: orthogonal projections to latent structures discriminant analysis
PCA: principal component analysis
PC: prostate cancer
PSA: prostate‐specific antigen
TUR‐P: transurethral resected prostate

## 1 Introduction

Bone metastatic disease is the lethal end‐stage of aggressive prostate cancer (PC). Patients with metastatic PC are generally treated with androgen‐deprivation therapy (ADT). This initially reduces metastasis growth, but after some time, castration‐resistant prostate cancer (CRPC) develops. Although several new treatments for CRPC have become available, they only temporarily retard disease progression (Omlin *et al*., 2014). Therapy selection in individual patients as well as future therapeutic developments need to be guided by deeper understanding of bone metastasis biology. This can probably not be obtained by studying primary tumors only or metastases at other locations, since metastases phenotypically diverge due to clonal expansions under the profound influence of different microenvironments, resulting in site‐dependent responses to treatment (Bergström *et al*., 2016; Van Etten and Dehm, 2016).

By exploring the transcriptome and proteome of bone metastases from patients, we have identified marked differences between metastases and primary tumors and, furthermore, identified bone metastasis subgroups of apparent biological importance (Djusberg *et al*., 2017; Hörnberg *et al*., 2011; Iglesias‐Gato *et al*., 2018; Jernberg *et al*., 2013; Nordstrand *et al*., 2018; Ylitalo *et al*., 2017). Based on gene expression of canonically androgen receptor (AR) regulated genes, 80% of the examined PC bone metastases were defined as AR‐driven and 20% were defined as non‐AR‐driven (Ylitalo *et al*., 2017). AR‐driven bone metastases had high sterol biosynthesis, amino acid and fatty acid degradation, and nucleotide biosynthesis (Ylitalo *et al*., 2017), while non‐androgen‐driven metastases showed high immune cell (Ylitalo *et al*., 2017) and bone cell activities (Nordstrand *et al*., 2018). Proteomic analysis identified two molecular subtypes of bone metastases with different phenotypes and prognosis (Iglesias‐Gato *et al*., 2018). These observations suggest possibilities for subtype‐related treatment of bone metastatic PC.

The current study made use of the largest set of clinical PC bone metastases published so far, with the specific aim to analyze gene expression profiles in relation to morphology and clinical parameters. The overall purpose of the study was to explore molecular variability in bone metastases that could be translated into strategies for improved treatment of metastatic PC.

## 2 Materials and methods

### 2.1 Patient samples

Samples of bone metastases were obtained from a series of fresh frozen and formalin‐fixed paraffin‐embedded biopsies collected from patients with PC operated for metastatic spinal cord compression at Umeå University Hospital (2003–2013). The patient series and the tissue handling have been previously described (Crnalic *et al*., 2010; Hörnberg *et al*., 2011; Ylitalo *et al*., 2017). Clinical and pathological characteristics of patients included in the current study are summarized in Table 1. In brief, most patients were diagnosed with locally advanced or metastatic disease, high serum prostate‐specific antigen (PSA) levels, and poor tumor differentiation [high Gleason score (GS)]. In patients where PC was not diagnosed until it caused neurological symptoms (patients without ADT at metastasis surgery), the primary tumor was not biopsied. Most patients were directly treated with ADT, while 10 patients had been previously treated with curative intent. In 52 cases (72%), there were available primary tumor biopsies for morphological analysis. At relapse to castration resistance, patients had been given second‐line treatments as indicated. Patients gave their informed consent, and the study was conducted in accordance with the Declaration of Helsinki. The study was approved by the local ethic review board of Umeå University (Dnr 03‐158, Dnr 04‐26M, Dnr 2013‐372‐32M).

**Table 1:** Patient characteristics at PC diagnosis and at time for metastasis  surgery in relation to metastasis  subtypes MetA‐C^a^. PSA, prostate‐specific antigen

	MetA^a^	MetB^a^	MetC^a^
	*n = *51	*n = *12	*n = *9
Age diagnosis (years)	71 (66; 76)	64 (59; 76)	71 (63; 76)
Age metastasis surgery (years)	74 (69; 80)	68 (62; 76)^*P = *0.084^	74 (71; 79)
PSA diagnosis (ng·mL^−1^)	160 (58; 920)	45 (19; 76)*	81 (29; 130)^*P = *0.075^
PSA metastasis surgery (ng·mL^−1^)	470 (110; 1100)	84 (44; 330)^*P = *0.059^	120 (110; 180)^*P = *0.068^
Follow‐up from diagnosis (months)	56 (29; 84)	30 (24; 65)	43 (30; 110)
Follow‐up from first ADT^b^ (months)	54 (25; 78)	30 (21; 43)	43 (30; 98)
Follow‐up from metastasis surgery (months)	10 (3; 33)	5 (2; 11)	13 (5; 19)
Gleason score at diagnosis			
7	13 (25%)	3 (25%)	3 (33%)
8	13 (25%)	2 (17%)	4 (44%)
9	10 (20%)	3 (25%)	1 (11%)
Not available	15 (29%)	4 (33%)	1 (11%)
Treatment with curative intention			
Radical prostatectomy	1 (2%)	0 (0%)	1 (11%)
Radiation	3 (6%)	4 (33%)**	1 (11%)
Previous ADT^b^			
None	9 (18%)	1 (8%)	2 (22%)
Short term^c^	4 (8%)	0 (0%)	0 (0%)
Long term	38 (74%)	11 (92%)	7 (78%)
Additional therapies			
Bicalutamide	17 (33%)	8 (67%)*	5 (56%)
Chemotherapy	4 (8%)	4 (33%)*	1 (11%)
Ra223	3 (6%)	1 (8%)	1 (11%)
Bisphosphonate	5 (10%)	1 (8%)	1 (11%)
Radiation toward operation site	7 (14%)	1 (8%)	1 (11%)
Soft tissue metastases	9 (18%)	5 (42%)^*P = *0.072^	1 (11%)^*P = *0.053^
Cancer cells^d^ (%)	70 (60; 80)	70 (70; 80)	50 (35; 50)**

Continuous variables given as median (25th; 75th percentiles), *P < 0.05; **P < 0.01, compared to MetA. ^a^Metastasis subtype, MetA‐C, as determined from PCA of whole genome expression profiles followed by unsupervised clustering (see Materials and methods for details). ^b^ADT included surgical ablation or LHRH/GnRH agonist therapy. ^c^ADT for 2–17 days before metastasis surgery. ^d^Fraction of cancer cell content in frozen metastasis sections extracted for RNA and analyzed by whole genome expression analysis.

Samples were also obtained from a historical cohort diagnosed with PC (1975–1991) at transurethral resection of the prostate (TUR‐P; Josefsson *et al.,* 2005, 2012). Patients with symptoms of metastatic disease at diagnosis and instantly treated with ADT were included.

### 2.2 Gene expression analysis

Whole genome expression analysis had been previously performed as two separate studies using the human HT12 Illumina Beadchip technique (Illumina, San Diego, CA, USA) version 3 in Hörnberg *et al*. (2011) and version 4 in Ylitalo *et al*. (2017). Here, bead chip data (GEO Datasets GSE29650 and GSE101607) were combined for all probes with average signals above two‐times the mean background level in at least one sample per study. Arrays were individually normalized using the quantile method. In addition, the data were centered by the mean for each probe, which completely removed batch effects (results not shown). Normalized datasets were merged by mapping Illumina ID and Hugo gene symbols. Redundant transcript probes were removed by selecting the probe with the highest median expression, leaving 10 784 gene transcripts for subsequent analysis. When merging bead chip data with RNA sequencing data (Quigley *et al*., 2018) for class discriminant analysis (below), data were centered by dividing intensities for each gene product by the median in each cohort.

### 2.3 Multivariate data analysis

Principal component analysis (PCA) was used to get an overview of the variability in data and to detect potential subgroups by unsupervised pattern recognition. Sevenfold cross‐validation testing was used to assess the reliability of the model. Cluster analysis was performed based on all genes or the first *m* (*m *=* *2, 5) principal components, using five clustering algorithms: (a) hierarchical clustering using the Euclidean distance and Ward linkage, (b) hierarchical clustering using the Manhattan distance and Ward linkage, (c) k‐means clustering, (d) self‐organizing maps, and (e) affinity propagation (Frey and Dueck, 2007).

A prediction model for subtype was built using orthogonal projections to latent structures discriminant analysis (OPLS‐DA; Bylesjö *et al*., 2006), based on levels for the top 20 gene products differentiating one sample cluster from the others (defined by the lowest *P* values in Mann–Whitney *U*‐test and a median fold change ≥ 1.5), and applied to an external validation cohort including 43 bone metastases from CRPC patients (Quigley *et al*., 2018). OPLS‐DA maximizes the explained variation in data (X) and its covariation with class membership, Y, defined by a dummy matrix of zeros and ones, and class membership was defined as default by predicted value (a) < 0.35 do not belong to the class, (b) between 0.35 and 0.65 intermediate, and (c) above 0.65 belong to the class. Multivariate data modeling was performed with simca software version 15.0 (Umetrics AB, Umeå, Sweden).

### 2.4 Functional enrichment analysis

Gene set enrichment analysis (GSEA) was performed by the metacore software (GeneGo, Thomson Reuters, New York, NY, USA). Analysis was based on gene transcripts significantly increased in one cluster compared to the others, as defined by Kruskal–Wallis followed by Mann–Whitney *U*‐test and adjusted *P* values (False Discovery Rate, < 0.01). Sets of genes associated with a functional process (pathway map or network) were determined as significantly enriched per subtype based on *P* values representing the probability for a process to arise by chance, considering the numbers of enriched gene products in the data vs. number of genes in the process. *P* values were adjusted by considering the rank of the process, given the total number of processes in the metacore ontology. Possible drivers of each subtype were identified by exploring the relations between subtype‐enriched transcripts and upstream regulators defined from the literature. *P*‐values were calculated for connectivity ratios between actual and expected interactions with objects in the data.

### 2.5 Metastases and primary tumor morphology

The fraction of tumor epithelial cells in metastasis tissue was determined using stereological techniques, as earlier described (Halin *et al*., 2007). Metastasis cell atypia was graded either as moderate or pronounced, and glandular differentiation was scored as observed or not. Cancer cells in metastases and primary tumor biopsies were immunostained and scored for AR, PSA, Ki67, and chromogranin‐A as earlier described (Crnalic *et al*., 2010). The PSA and AR staining were quantified using a scoring system based on the percentage (0: 0%, 1: 1–25%, 2: 26–50%, 3: 51–75%, and 4: 76–100%) and intensity (0: negative, 1: week, 2: moderate, and 3: intense staining) of immunostained tumor epithelial cells. An immunoreactivity (IR) score was obtained by multiplying the scores for distribution and intensity, giving IR scores in the range of 0–12. Ki67 and chromogranin‐A staining was quantified as the percentage of stained tumor epithelial cells. The stroma in primary tumor biopsies was scored for the percentage of AR‐positive cells as earlier described (Wikström *et al*., 2009) and for a reactive desmoplastic response, characterized by loss of stroma smooth muscle and increase in fibroblasts and collagen, using a 3‐tier scoring system (Saeter *et al*., 2015).

### 2.6 Univariate statistics and survival analysis

For continuous variables, groups were compared using the Kruskal–Wallis *H*‐test followed by the Mann–Whitney *U*‐test. Paired samples were analyzed using the Wilcoxon test. Correlations between variables were analyzed using Spearman rank test. The chi‐square test was used for categorical values. Survival analysis was performed by Kaplan–Meier analysis with death of PC as event and death by other causes as censored events and with follow‐up time considering time from first ADT until the latest follow‐up examination (March 2017). Statistical analyses were performed using the Statistical Package for the Social Sciences, spss 24.0 software (SPSS, Inc, Chicago, IL, USA).

## 3 Results

### 3.1 Global gene expression in bone metastases and identification of molecular subtypes

**Figure 1:**
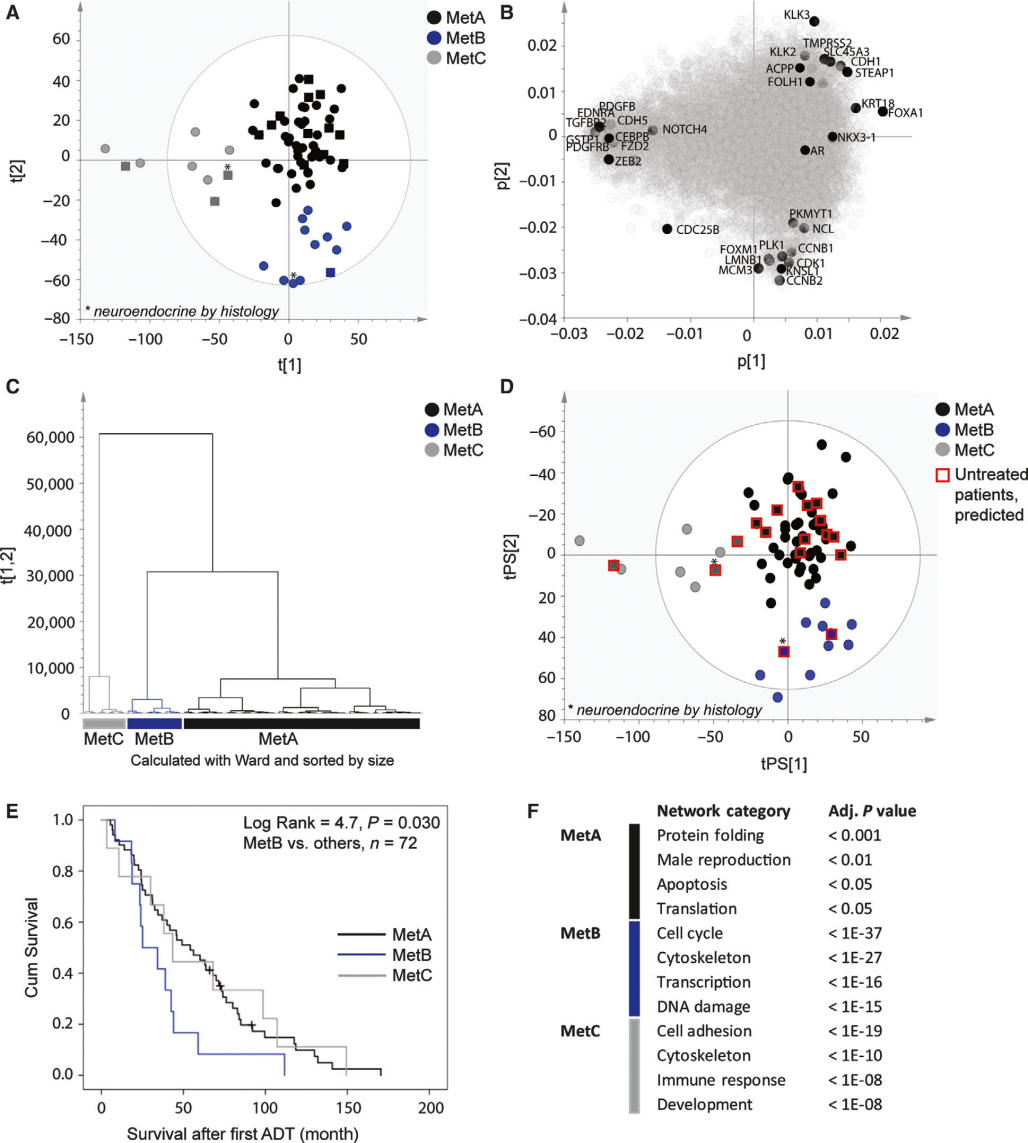
PCA and unsupervised clustering of 72 bone metastasis samples, based on whole genome expression analysis (Illumina bead chip array), identify three main clusters of samples: MetA, MetB, and MetC. Score plot (A) and loading plot (B) showing MetA‐C in black, blue, and gray, respectively, based on the two‐first principal components and the clusters in (C). Samples from CRPC patients are represented by circles, and samples from nontreated and short‐term castrated patients are shown as squares. Two neuroendocrine metastases are indicated by stars. Selected sets of gene products enriched in the MetA‐C clusters are highlighted. (D) Predictions of nontreated, short‐term treated, and neuroendocrine samples (red squares) into clusters defined from PCA of CRPC samples only. (E) Kaplan–Maier plot showing poor cancer‐specific survival for MetB patients after ADT. (F) Top four enriched network categories per Met subtype, according to GSEA using the metacore software (for complete lists of functionally enriched networks, see Table S2).

The global gene expression pattern in 12 treatment‐naïve, four short‐term castrated, and 56 CRPC bone metastases was explored. Based on transcript levels of 10 784 nonredundant genes, a PCA model was built that included nine significant principal components explaining 40% of the variation in the data (Table S1). Hierarchical cluster analysis using the Euclidean distance and the first two principal components revealed three molecular subtypes of bone metastases, referred to as bone metastasis subtypes A, B, and C (MetA‐C; Fig. 1). The majority of samples clustered as MetA (71%), while 17% and 12% clustered as MetB and MetC, respectively (Fig. 1A–C), based on the loadings (gene expression levels) in Fig. 1C. The inclusion of five principal components and the use of alternative clustering methods verified robust clustering with preserved grouping of 90% of the samples, and 90%, 83%, and 100% consistency for the MetA, MetB, and MetC samples, respectively (Fig. S1). Alternative clustering methods using all genes preserved 97%, 93%, 92%, and 82% of the group belongings for k‐means, hierarchical clustering (Euclidean), hierarchical clustering (Manhattan), and self‐organizing map, respectively, while the affinity propagation algorithm was unable to converge.

The MetA‐C clusters were identified also when data analysis was based on CRPC samples only (Fig. 1D), leaving samples from treatment‐naïve and short‐term castrated patients outside the PCA modeling together with two CRPC samples defined as neuroendocrine (NE, based on high chromogranin‐A and low PSA, AR expression). Those samples were predicted with 100% consistency, and previously untreated metastases were identified within all clusters (Fig. 1D), indicating that the MetA‐C subtypes could be intrinsic and not only developed by the introduction of castration therapy.

To enable validation of the MetA‐C subtypes in an external cohort of bone metastases from CRPC patients (Quigley *et al*., 2018), the 20 most differentiating gene products per subtype were identified and used for PCA and OPLS‐DA modeling (Fig. S2). Expression levels for the MetA‐, MetB‐, and MetC‐differentiating genes, respectively, were highly correlated also within the validation cohort and responsible for differentiating samples into three clusters (Fig. S2A–F). Accordingly, the MetA‐C subtypes in the validation cohort were predicted at frequencies comparable to those originally observed (Fig. S2G–J).

### 3.2 Metastasis subtypes relate to patient characteristics and prognosis

To assess the clinical relevance of the molecular subtypes, MetA‐C were analyzed in relation to patient characteristics in Table 1. Patients with the MetB subtype had shorter cancer‐specific survival after ADT than MetA and MetC patients (median survival 25 vs. 49 months, respectively, *P = *0.030, Fig. 1E), and lower serum PSA levels compared to MetA patients at diagnosis (0.28‐fold, *P = *0.011) and borderline at Met surgery (Table 1). A tendency of low PSA levels was seen also in MetC patients (Table 1). As described above, the subtypes were apparently not related to previous ADT (Fig. 1), while a relatively high proportion of MetB patients had undergone radiation therapy to primary tumor (*P = *0.006) and received bicalutamide and/or chemotherapy subsequent to ADT (*P = *0.038 and 0.017, respectively, Table 1). Neither primary tumor GS nor patient age or presence of soft tissue metastases were significantly associated with any specific subtype.

### 3.3 Metastasis subtypes have different morphology

**Figure 2:**
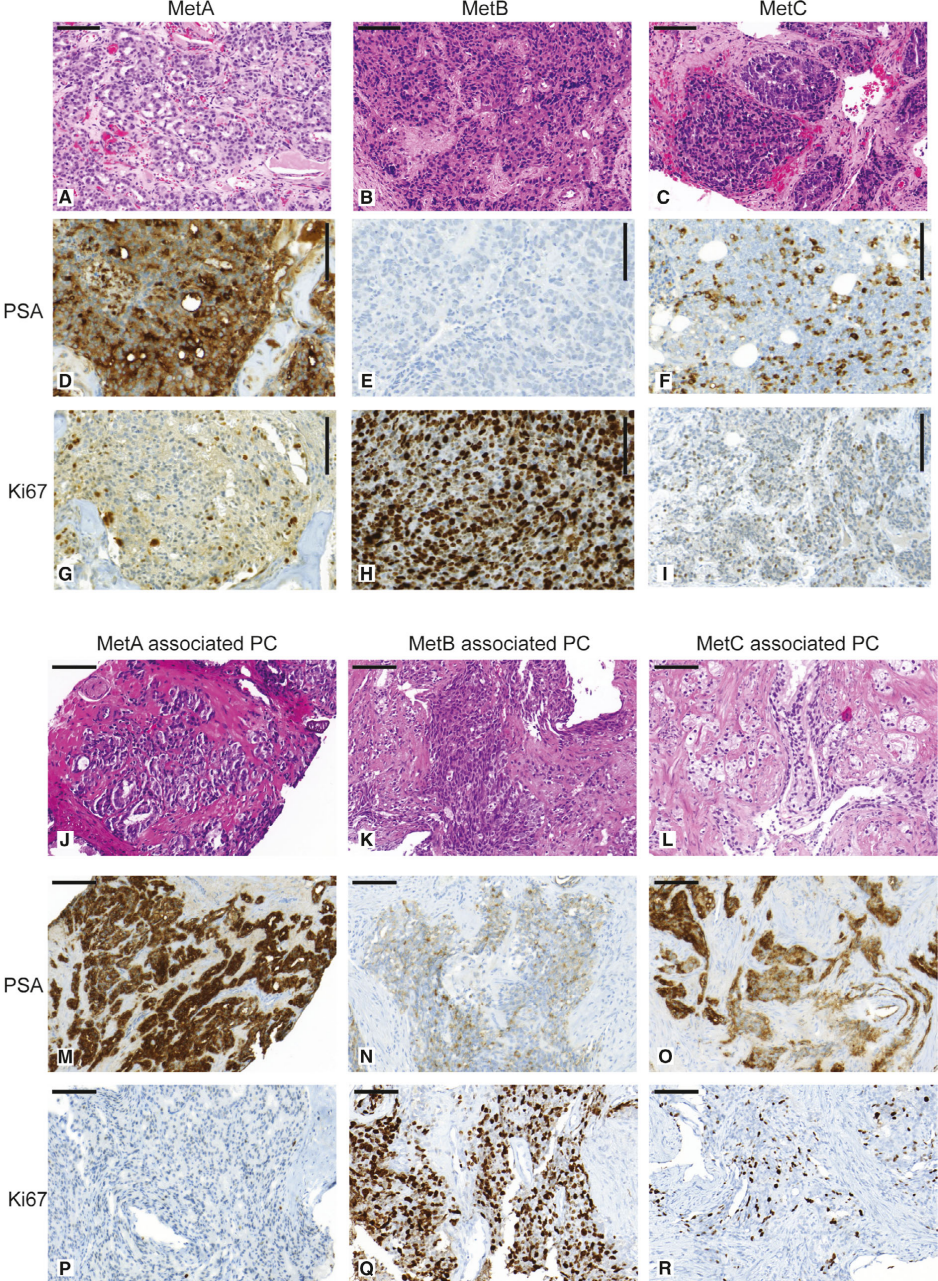
Representative tissue sections of MetA, MetB, and MetC bone metastases and associated primary tumors stained with HTX‐eosin (A–C) and (J–L), PSA (D‐F) and (M–O), and Ki67 (G–I) and (P–R). MetA is characterized by moderate cellular atypia, glandular differentiation, relatively low fraction of Ki67‐positive cells (proliferating cells) and high PSA IR. MetB shows prominent cellular atypia, lack of glandular differentiation, low PSA IR, and high tumor cell proliferation. MetC shows prominent cellular atypia with glandular differentiation detectable in some cases, low cell proliferation, relatively low tissue PSA IR, and relatively high stroma/epithelial ratio. MetA‐associated primary tumors are characterized by high PSA IR and relatively low proliferation. MetB‐associated primary tumors show low PSA IR, high proliferation, and a reactive stroma response. MetC‐associated primary tumors show relatively high proliferation with PSA IR and reactive stroma response intermediate between MetA and MetB cases. Bar indicates 100 μm.

Most metastases were poorly differentiated with sheets of tumor epithelial cells resembling Gleason grade 5, while some showed patterns similar to Gleason grade 4 (Fig. 2A–C). Some metastases showed a prominent connective tissue stroma (Fig. 2A–C). The fraction of cancer cells was significantly lower in MetC compared to MetA tumor sections (Table 1). Importantly, this was seen both in the frozen sections (used for gene expression analysis) and in the paraffin‐embedded tissue (used for morphology analysis) representing distinct metastasis areas from the same patient, suggesting intrinsic differences in epithelium/stroma ratio between subtypes. Additional subtype‐related differences were identified based on histological and immunohistochemical (IHC) analysis of markers previously associated with aggressive PC (summarized in Table 2), with the most pronounced being reduced tissue PSA, increased proliferation (fraction of Ki67‐stained tumor cells), cellular atypia, and lack of glandular structures in MetB. Marked intratumor heterogeneity in immune‐staining pattern was observed, as previously reported (Crnalic *et al*., 2010).

**Table 2:** Molecular metastasis subtypes MetA‐C in relation to metastasis and primary tumor morphology. Continuous variables given as median (25th; 75th percentiles).

	MetA (*n = *51)	MetB (*n = *12)	MetC (*n = *9)
Bone metastases			
AR score (0–12)	8 (4;12)	10 (6;12)	9 (4;12)
	(*n = *49)	(*n = *12)	(*n = *8)
PSA score (0–12)	9 (6;12)	2 (1;6)***^a^	6 (1;9)*^a^
	(*n = *51)	(*n = *12)	(*n = *9)
Ki67 (%)	14 (9;20)	33 (22; 45)***^a^	12 (8;28)*^b^
	(*n = *50)	(*n = *12)	(*n = *9)
Chromogranin‐A (%)	0 (0;0.2)	0.2 (0;1.6)^*P = *0.05^ ^a^	0 (0;0)
	(*n = *45)	(*n = *12)	(*n = *9)
Cellular atypia (moderate; high)	34; 17	2; 10**^a^	2; 7*^a^
Gland formation (yes; no)	20; 31	0; 12**^a^	4; 5*^a^

	MetA associated (*n = *36)	MetB associated (*n = *8)	MetC associated (*n = *8)
Primary tumor			
AR score (0–12)	12 (12;12)	12 (10;12)	10.5 (8;12)
	(*n = *34)	(*n = *8)	(*n = *8)
PSA score (0–12)	9 (8;12)	6 (4;8)**^a^	7 (6;10.5)
	(*n = *32)	(*n = *8)	(*n = *8)
Ki67 (%)	9 (6.5;14)	19 (15;26)**^a^	17 (11;30)*^a^
	(*n = *35)	(*n = *8)	(*n = *8)
AR tumor stroma score (% of stroma cells positive)	22 (15;30)	11 (4;17)*^a^	17 (7;20)
	(*n = *34)	(*n = *8)	(*n = *8)
Reactive stroma score (1; 2; 3)	11; 11; 0	0; 2; 7***^a^	0; 6; 1*^a,^ *^b^

Metastasis subtype, MetA‐C, as determined from PCA of whole genome expression profiles followed by unsupervised clustering (Fig. 1). *P < 0.05, **P < 0.01, ***P < 0.001, ^a^significantly different from MetA, ^b^significantly different from MetB.

### 3.4 Enrichment of divergent functional processes per metastasis subtype

To identify subtype‐enriched functional processes, gene transcripts with significantly increased levels per subtype were subjected to GSEA in the metacore software. Network analysis showed enrichment of protein translation and folding, male reproduction, and regulation of apoptosis in MetA; cell cycle and DNA damage response, cytoskeleton reorganization, and transcription in MetB; and cell adhesion, cytoskeleton, immune response, and development in MetC (Fig. 1F, Table S2). Pathway analysis demonstrated enrichment of ‘AR activation and downstream signaling in PC’ in MetA compared to other subtypes (Table S3), based on high transcript levels of *KLK3* and other canonically AR‐regulated genes such as *KLK2, FOLH1, STEAP1, TMPRSS2, SLC45A3, ACPP (PPAP),* and *CDH1* (Figs 1B and S3). MetA also showed high expression of the luminal cell marker *KRT18* (Fig. 1B) and enrichment of metabolic pathways involving amino acid and fatty acid degradation (Table S3). The MetB subtype showed pathway enrichment representing all phases of the cell cycle (Table S3), including ‘Initiation of mitosis,’ based on high *FOXM1, CCNB1, CCNB2, CDC25B, CDK1, PLK1, PKMYT1, LMNB1, KNSL1, and NCL* expression (Figs 1B and S4). Other markedly enriched pathways in MetB included response to DNA damage and transcription (Table S3). MetB expression levels of *KRT18* were similar to MetA, while most luminal cell markers like as *KLK3* and *CDH1* were reduced, indicating luminal cell dedifferentiation coupled to increased cell division. Among many enriched pathways in MetC, ‘ECM remodeling,’ ‘regulation of EMT,’ and ‘immunological synapse formation’ were among the most prominent (Table S3). Enrichment of ‘the EMT pathway’ in MetC was based on high levels of transcripts involved in Wnt, Notch, TGF‐beta, and PDGF signaling (Figs 1B and S5). MetC showed low expression of luminal cell markers, but was enriched for some transcripts indicating a basal cell phenotype, that is, *CEBPB* and *GSTP1*. Other basal cell markers like *p63* and *CK5* were low in all cases. Expression levels of luminal cell markers *AR* and *NKX3.1* did not significantly differ between subtypes.

### 3.5 Possible drivers of metastasis subtypes

As the MetB subtype was associated with the worst clinical outcome, we tried to identify putative drivers of its key characteristics, that is, luminal cell dedifferentiation and proliferation. Based on connectivity analysis of gene networks and upstream regulators, a set of interesting candidate drivers were identified, such as the *FOXA1* transcription factor (*HNF3alpha*) in MetA and the *FOXM1* transcription factor in MetB (Table S4). While FOXA1 may interact with the AR in MetA to drive canonical AR signaling and luminal differentiation (Figs 1B and S3), FOXM1 may drive proliferation in MetB (Figs 1B and S4; Wierstra and Alves, 2007). Several kinases with inhibiting drugs available in the clinic for treatment of other cancer types or in clinical trials were suggested as upstream regulators for specific subtypes, for example, ErbB2 (MetA), AURORA A/B (MetB), and PDGF‐R‐beta (MetC; Table S4), hypothetically indicating possibilities for developing subtype‐related therapeutic strategies.

### 3.6 Immunohistochemistry to determine metastasis subtype

**Figure 3:**
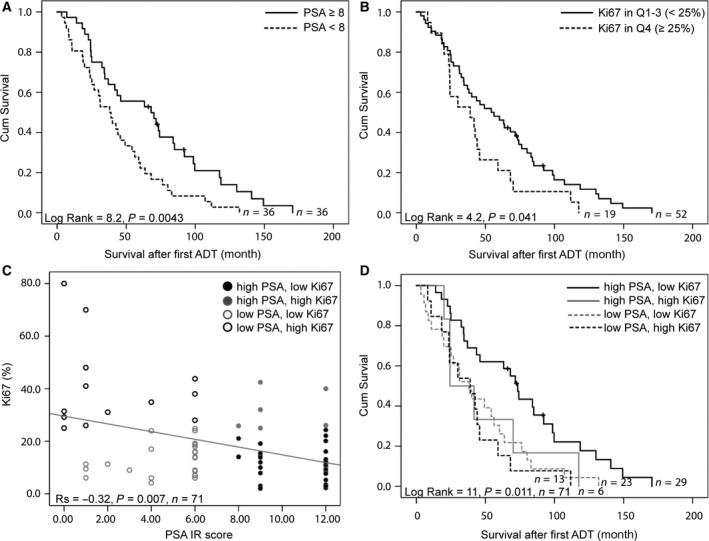
Kaplan–Meier analysis of PSA IR score and proliferation rate (fraction of Ki67‐stained tumor cells) in metastasis samples in relation to cancer‐specific survival after treatment with ADT. PSA IR was dichotomized as above (high) or below (low) median and Ki67 as quartile 4 (high) or below (low) (A–B). A combinatory PSA and Ki67 score was obtained based on their inverse correlation and the cut‐offs used in A–B (C). Patients with high PSA, low Ki67 metastases show the best prognosis with significantly longer cancer‐specific survival after first ADT than other patients (D).

Based on gene expression data and morphological observations, PSA and Ki67 were selected as potential surrogate markers for MetA and MetB, respectively (Fig. 2D–I, Table 2). Notably, the median PSA staining score was higher in metastases with than without glandular differentiation (9 vs. 6, *P = *0.016, *n = *72) and in cases without pronounced atypia (9 vs. 6, *P = *0.012, *n = *72), suggesting that high cellular PSA is a marker for preserved epithelial and glandular differentiation in tumor cells. Accordingly, patients with low PSA staining scores (below median, scores 0–6) and high proliferation (fraction of Ki67‐stained cells in the upper quartile, ≥ 25%), respectively, had short cancer‐specific survival after first ADT in comparison with other patients (Fig. 3A–B). The PSA staining score inversely correlated to tumor cell proliferation in bone metastases (*R*_s_ **=* *−0.32, *P = *0.007, *n = *71; Fig. 3C), and a combinatory score identified four groups of metastases with the following frequencies: high PSA, low Ki67 (41%); low PSA, low Ki67 (32%); low PSA, high Ki67 (18%); high PSA, high Ki67 (8.5%) (Fig. 3D). Patients with high PSA, low Ki67 metastases showed the best prognosis (Fig. 3D) and were enriched for MetA samples (86%). MetB samples were enriched among the low PSA, high Ki67 samples (69%), whereas MetC was not specifically enriched by these markers.

### 3.7 Comparisons between bone metastases and paired primary tumors

We then examined whether subtype‐related difference in metastases could be traced back to the corresponding primary tumors, by exploring morphologic factors in diagnostic needle biopsies, as summarized in Table 2 and demonstrated in Fig. 2J–R. Collectively, these observations indicated that characteristics of MetB, such as high proliferation and low tissue PSA, may be detectable already in the primary tumor (Table 2). Primary tumors of MetB patients also showed low AR staining in the tumor stroma coupled to a reactive stroma response (Table 2).

Pairwise analysis showed significantly reduced AR (*P = *2.3E‐5, *n = *34) and PSA (*P = *0.017, *n = *32) staining in MetA metastases compared to their corresponding primary tumors, while the fraction of Ki67‐positive cells was significantly increased (*P = *0.013, *n = *35; Fig. S6). Similar trends were seen for those markers in MetB or MetC patients, although no significant changes were observed possibly due to the low number of pairs in those groups (Fig. S6).

### 3.8 Determining prognosis by analysis of subtype‐related markers in primary tumors

**Figure 4:**
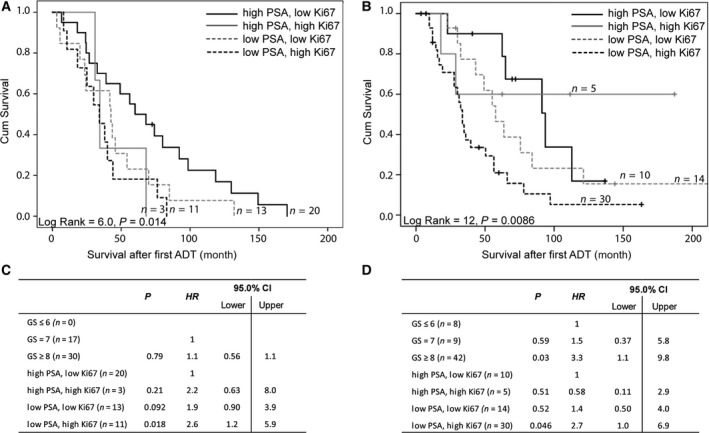
Kaplan–Meier analysis of combinatory PSA and Ki67 IR scores of primary tumor samples in relation to cancer‐specific survival after treatment with ADT in metastatic MetA‐C patient cohort (A) and in a validation cohort of TUR‐P‐diagnosed patients (B). PSA IR was dichotomized as above (high) or below (low) median and Ki67 as quartile 4 (high) or below (low), using cutoff values for the corresponding cohorts. (A) Patients with high PSA, low Ki67 primary tumor IR show significantly longer cancer‐specific survival after first ADT than other patients. (B) Patients with high PSA, low Ki67 show longer and patients with low PSA, high Ki67 show shorter cancer‐specific survival after first ADT compared to other patients. (C–D) Multivariate Cox analysis shows that the combinatory PSA, Ki67 IR scores of primary tumors add prognostic value to GS in metastatic (C) and TUR‐P (D) patient cohorts.

We further explored whether surrogate IHC markers for the MetA and MetB phenotypes could differentiate patient outcome also if analyzed in primary tumor tissue. High Ki67 and low PSA IR (MetB characteristics) were associated with short survival after first ADT in two different cohorts; (a) primary tumor biopsies of the MetA‐C patients in the current study (Fig. 4A) and (b) TUR‐P‐diagnosed cases (Fig. 4B), when using the PSA median and Ki67 upper quartile as cutoff values within each cohort. Patients with the combination of high PSA and low Ki67 (MetA characteristics) had a more favorable outcome than other patients when treated by ADT (Fig. 4A–B). The combinatory PSA and Ki67 IR score provided independent prognostic information to GS in multivariate survival analysis (Fig. 4C–D).

## 4 Discussion

This study suggests the existence of molecular subtypes of PC bone metastases, here named MetA, MetB, and MetC. The MetA‐C subtypes are related to morphological and phenotypic tumor characteristics and to patient outcome after ADT, and we therefore suggest them to be of high clinical significance. Importantly, the MetA‐C are predicted at frequencies comparable to those originally observed also in the second largest publicly available bone Met cohort (43 cases; Quigley *et al*., 2018).

The most common metastasis subtype (MetA) seems to be of luminal cell origin, according to expression of luminal cell differentiation markers and androgen‐stimulated genes, including *KRT18, FOXA1* and *KLK3* (PSA), and signs of glandular differentiation and low cell proliferation. MetA patients have high serum PSA levels and show a favorable prognosis after ADT. The phenotype of MetA thus resembles that of luminal prostate epithelium, an assumption supported by PCA showing similar gene expression profiles in radical prostatectomy and MetA samples (Fig. S7).

The second most common subtype (MetB) shows poor prognosis after ADT and some features similar to neuroendocrine tumors, such as high cell cycle activity and DNA damage (Flores‐Morales *et al*., 2019), but as chromogranin expression is generally low and *KRT18* and AR expression retained, we suggest that MetB shows a dedifferentiated luminal phenotype. The contrasting processes of cell differentiation and proliferation are both driven by androgens in the prostate (Cai *et al*., 2011; Gao *et al*., 2016; Yang *et al*., 2016), but in a context‐dependent way that seems reprogrammed during cancer progression by coactivators and corepressors modulating the AR cistrome (Sharma *et al*., 2013; Liu *et al*., 2017). AR activation in the presence of coactivator FOXA1 results in cell differentiation, PSA secretion, and suppressed proliferation (Cai *et al*., 2011; Gao *et al*., 2003, 2016; Yang *et al*., 2016), while in cells with low FOXA1, this instead stimulates cell proliferation (Yang *et al*., 2016). In the MetB subtype, androgen‐stimulated gene expression is generally low, tumor cells are dedifferentiated, and cell proliferation is high, in parallel with transcript levels of the proliferation‐associated transcription factor FOXM1. FOXM1 is known to initiate mitosis (Wierstra and Alves, 2007), and FOXM1 inhibition has been shown to retard tumor growth in a model system for the PCS1 subtype (Ketola *et al*., 2017). In the current study, approximately 13% of the samples showed an intermediate subtype with characteristics of both MetA and MetB, and in the validation cohort (Quigley *et al*., 2018), this was observed in about 14%. In the LNCaP cell line, single‐cell sequencing has demonstrated the existence of multiple subclones where some appear similar to MetA, whereas others are more MetB‐like with high cell proliferation and reduced androgen dependency (Horning *et al*., 2018). Collectively, this suggests that the luminal‐derived MetA subtype may be able to dedifferentiate in to the more aggressive MetB subtype, possibly driven by altered AR‐activity.

The less common and poorly defined subgroup MetC is identified based on enrichment of transcripts involved in stroma–epithelial interactions such as cell adhesion, cell and tissue remodeling, immune responses, and inflammation. Processes in MetC thus resemble those previously described by us for non‐AR‐driven bone metastases (Nordstrand *et al*., 2018; Ylitalo *et al*., 2017). One suggested upstream regulator of MetC is the C/EBP transcription factor, generally associated with inflammation and downregulated by AR signaling (Barakat *et al*., 2015). C/EBP is anti‐apoptotic and causes chemoresistance in CRPC, and thus constitutes a potential therapeutic target (Barakat *et al*., 2015). The stroma fraction in MetC is higher than in MetA, and although this is repeatedly observed in separate metastases of MetC patients, it remains to be shown to what extent the molecular characteristics of MetC are only a consequence of lower epithelial content or a key marker of a clearly different tumor cell phenotype. Furthermore, the cellular origin of MetC and surrogate markers for this apparently multifaced metastasis phenotype remains to be discovered. To do this, a larger cohort of MetC cases is needed.

Importantly, we here demonstrate that morphological characteristics of bone metastasis subtypes can be traced back to their corresponding primary tumors (see below). In line with this, the MetA, MetB, and MetC subtypes show phenotypic characteristics resembling those previously described for molecular subtypes of primary prostate tumors: the PC subtypes 2, 1, and 3 (You *et al*., 2016) and the luminal A, luminal B, and basal subtypes as determined by the PAM50 breast cancer panel (Zhao *et al*., 2017), respectively. Taken together, this may suggest that the primary tumor subtype could be maintained in bone metastases. However, the increased tumor cell proliferation and decreased cellular PSA level in metastases compared to primary tumors together with their large difference in the global proteome (Iglesias‐Gato *et al*., 2018) argue for major differences between primary tumors and their corresponding metastases that need to be recognized. Accordingly, the top 60 differentiating gene products for MetA‐C show minor overlap with the biomarkers suggested to differentiate primary prostate tumors into molecular subtypes (You *et al*., 2016; Zhao *et al*., 2017) and with biomarkers on approved tests for predicting risk in patients with localized disease (Prolaris, OncotypeDx, GenomeDx) (Loeb and Ross, 2017), with a total overlap of only 5/60 (8%). Hypothetically, intrinsic properties and changes induced by the microenvironment and therapy could all influence subtype characteristics and explain both similarities and differences between primary tumors and their metastases that all need to be taken into consideration when developing novel treatments for metastatic PC.

The MetA and MetB subtypes show characteristics similar to the BM1 and BM2 subgroups, respectively, recently identified by proteome profiling of a subset of the bone metastases in the current study (Iglesias‐Gato *et al*., 2018). Two important routes separating those subtypes are obviously related to cell proliferation and cell differentiation, and the metastasis phenotype might thus be predicted by combined analysis of a few markers, similarly to what is regularly done in breast cancer (Duffy *et al*., 2017). IR for Ki67 (or MCM, see Iglesias‐Gato *et al*. (2018)) and PSA could serve as surrogate markers for cell proliferation and differentiation, respectively, and by that differentiating less aggressive MetA from more aggressive MetB. High proliferation and low tumor cell PSA synthesis have previously been linked to poor prognosis in both primary tumors (Bubendorf *et al*., 1998; Fisher *et al*., 2013; Josefsson *et al*., 2005, 2012; Stege *et al*., 2000) and bone metastases (Crnalic *et al*., 2010; Iglesias‐Gato *et al*., 2018) and recently used in combination to predict outcome in patients managed with watchful waiting (Hammarsten *et al*., 2019). Interestingly, the PAM50 biomarker panel originally developed for breast cancer subtyping identify clinically relevant subgroups also in a variety of other cancer types (Zhao *et al*., 2019), possibly because it mainly separates tumors according to key biological factors such as cell differentiation and proliferation. Future studies evaluating the prognostic usefulness of surrogate markers like combined PSA and Ki67 IR, possibly also in combination with additional subtype‐enriched markers including markers for MetC, are therefore warranted for both primary tumor and metastatic disease.

When the key molecular drivers for different metastasis subtypes have been defined, the ADT should perhaps be accompanied by subtype‐specific treatments. For MetA patients, ADT seems effective, but in this luminal subtype, androgen signaling could also have cell differentiating effects and, if so, ADT may have adverse effects and additional metabolic targeting could be an option. In MetB patients where ADT seems least effective, it should probably be complemented upfront with chemotherapy, or by direct targeting of tumor‐promoting factors driving the cell cycle or DNA repair. Patients with MetB bone metastases have reduced AR levels and morphological signs of a reactive stroma response already in their primary tumor stroma, something previously associated with poor response to ADT and a poor prognosis (Wikström *et al*., 2009). For those cases, stroma‐targeted therapies could be of interest. In breast cancer, responsiveness to hormonal therapy seems to be regulated by signals in the cancer stroma as stroma interfering was able to convert basal, hormone treatment‐resistant breast cancer into a luminal, treatment‐responsive subtype (Brechbuhl *et al*., 2017; Roswall *et al*., 2018). For MetC, potential therapeutic targets in the tumor microenvironment may be available, such as immune or bone cells. Further examination of subgroup‐associated differences in metastasis stroma is warranted.

## 5 Conclusions

Bone metastases in PC patients can be separated based on gene expression profiles into molecular subtypes (MetA‐C) with different morphology, phenotype, and outcome. The MetA‐C subtypes could probably be identified by analyzing a set of surrogate markers in metastasis tissue. Metastases with the best (MetA) and worst (MetB) prognosis seem predictable also from analyzing primary tumors. Underlying reasons for the development of the MetA‐C subtypes are currently unknown and need further attention, but may relate to basal/luminal cellular origin and/or genetic, epigenetic defects affecting differentiation and clonal expansion. To what extent the MetA‐C subtypes are intrinsic and predictable from the primary tumor or develop at the metastatic site under the influence of a different microenvironment and therapy cannot be concluded from the current study, but would need longitudinal monitoring within the same patient. Limitations of the study include the relatively low number of samples analyzed and the current lack of validation cohorts. Inter‐ and intrametastasis heterogeneity also needs to be considered (Crnalic *et al*., 2010; Roudier *et al*., 2003; Shah *et al*., 2004). The findings of the current study need to be verified in future bone metastasis  cohorts with clinical follow‐up data available. To test whether patients with different metastasis subtypes/phenotypes would benefit from different treatment strategies, prospective studies evaluating the MetA‐C subtype in relation to therapy response in patients given conceptually different treatments (i.e., AR targeting, chemotherapy, immunotherapy) as well as novel subtype‐specific therapies are needed.

## Conflict of interest

The authors declare no conflict of interest.

## Author contributions

ET, AB, and PW conceived and designed the study. EBY, EJ, AB, and PW acquired the data. ET, LV, AB, and PW analyzed and interpreted the data. ET, AB, and PW wrote the manuscript draft that all authors critically revised. SC, DIG, AM, PS, LE, and AW contributed with technical and material support. PR, AB, and PW supervised the study.

## Supporting information


**Fig. S1.** Summary of cluster analysis.
**Fig. S2.** Principal component analysis and orthogonal projections to latent structures discriminant analysis of bone metastasis samples in GEO Datasets GSE29650 and GSE101607 and in validation data set data (Quigley *et al*., 2018).
**Fig. S3.** Top functionally enriched pathway in metastasis subtype MetA.
**Fig. S4.** Functionally enriched pathway map in metastasis subtype MetB.
**Fig. S5.** The top two functionally enriched pathway map in metastasis subtype MetC.
**Fig. S6.** Paired observations of androgen receptor (AR), PSA, and Ki67 immunoreactivity scores in bone metastases of subtypes A‐C and in corresponding primary tumor biopsies.
**Fig. S7.** Predictive score plot for the principal component analysis of 72 bone metastasis and 13 non‐malignant prostate samples.

**Table S1**. Principal component analysis model summary.

**Table S2.** Enriched functional networks per metastasis subtype according to metacore software.

**Table S3.** Enriched functional pathways per metastasis subtype according to metacore software.

**Table S4.** Overconnecting gene products per metastasis subtype according to metacore software.

## Data Availability

Datasets GSE29650 and GSE101607 are available at https://www.ncbi.nlm.nih.gov/geo/
