# Supplementary material for: Gene expression profiles define molecular subtypes of prostate cancer bone metastases with different outcomes and morphology traceable back to the primary tumor
Source: Mol Oncol. 2019 Jun 27;13(8):1763–77. doi: 10.1002/1878-0261.12526 (PMC6670017; doi:10.1002/1878-0261.12526)
Supplement: Supplementary file 1 — Fig. S1. Summary of cluster analysis. Fig. S2. Principal component analysis and orthogonal projections to latent structures discriminant analysis of bone metastasis samples in GEO Datasets GSE29650 and GSE101607 and in validation data set data (Quigley et al., 2018). Fig. S3. Top functionally enriched pathway in metastasis subtype MetA. Fig. S4. Functionally enriched pathway map in metastasis subtype MetB. Fig. S5. The top two functionally enriched pathway map in metastasis subtype MetC. Fig. S6. Paired observations of androgen receptor (AR), PSA, and Ki67 immunoreactivity scores in bone metastases of subtypes A‐C and in corresponding primary tumor biopsies. Fig. S7. Predictive score plot for the principal component analysis of 72 bone metastasis and 13 non‐malignant prostate samples. [file MOL2-13-1763-s001.docx]

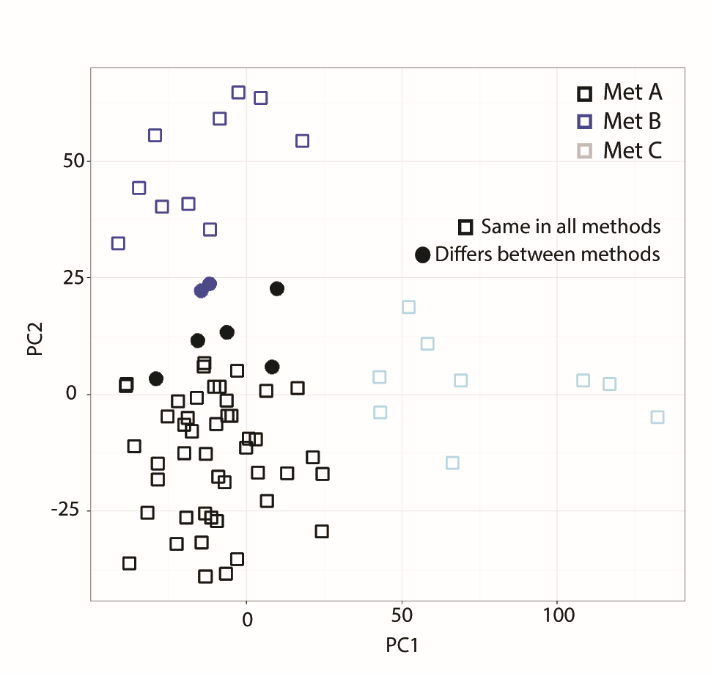


**Fig. S1.** Summary of cluster analysis, showing the two first principal components for the PCA analysis using five clustering algorithms: i) Hierarchical clustering using the Euclidian distance and Ward linkage, ii) Hierarchical clustering using the Manhattan distance and Ward linkage, iii) k-means clustering, iv) Self Organizing maps and v) Affinity propagation.


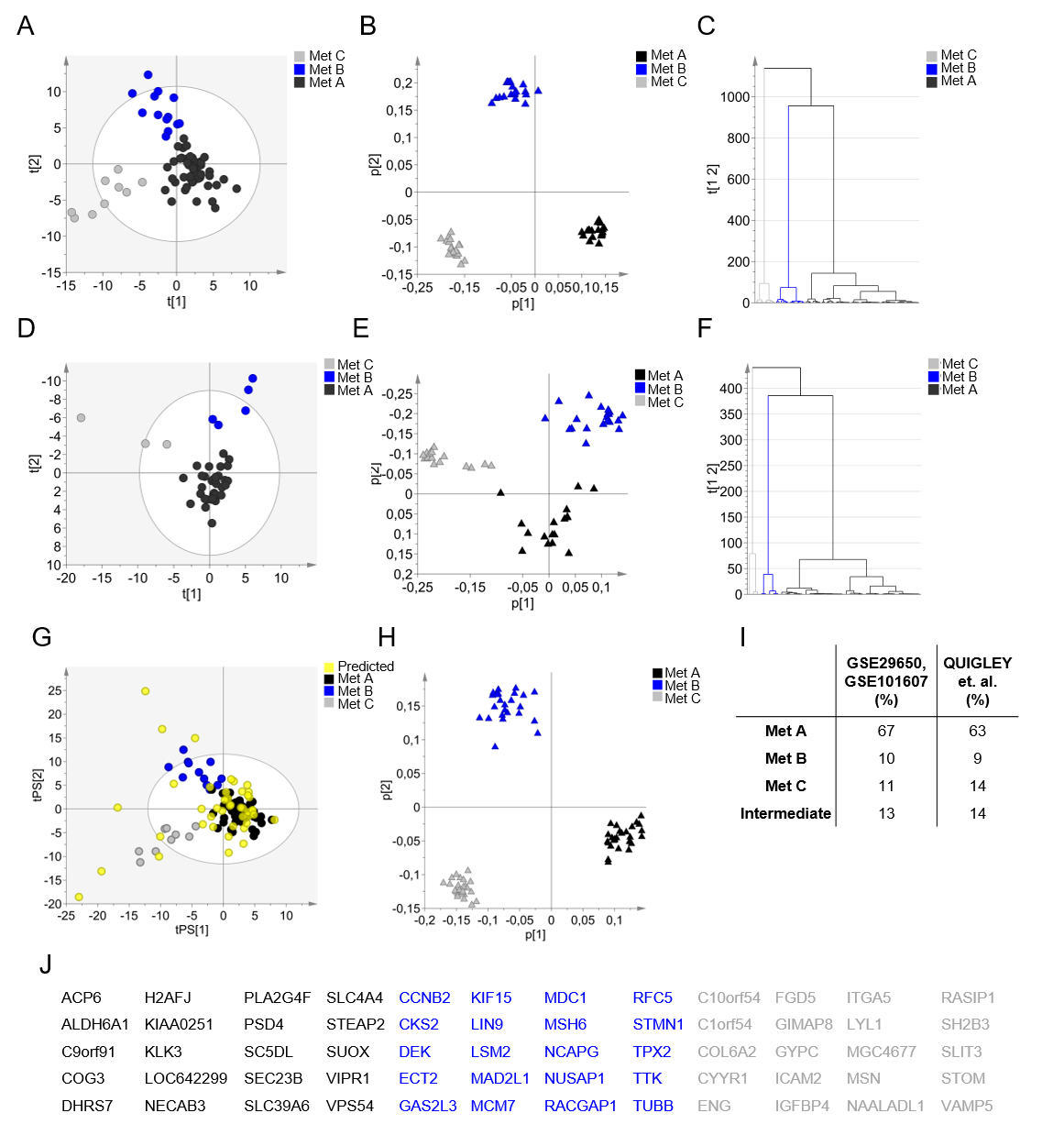


**Fig. S2.**  Principal component analysis (PCA) and orthogonal projections to latent structures discriminant analysis (OPLS-DA) of bone metastasis samples, based on gene expression levels of top 20 differentiating genes for each subtype showing the score plot, loading plot and HCA cluster analysis of A-C) PCA of GEO Datasets GSE29650 and GSE101607, D-F) PCA of RNA seq. data (Quigley *et al*, 2018), G-H) OPLS-DA model for MetA-C based on 72 samples (GSE29650 and GSE101607) and prediction of 43 external samples (yellow) (Quigley *et al*, 2018), giving frequencies as shown in table (I). J) List of top 20 differentiating genes per subtype (for details see material and methods).


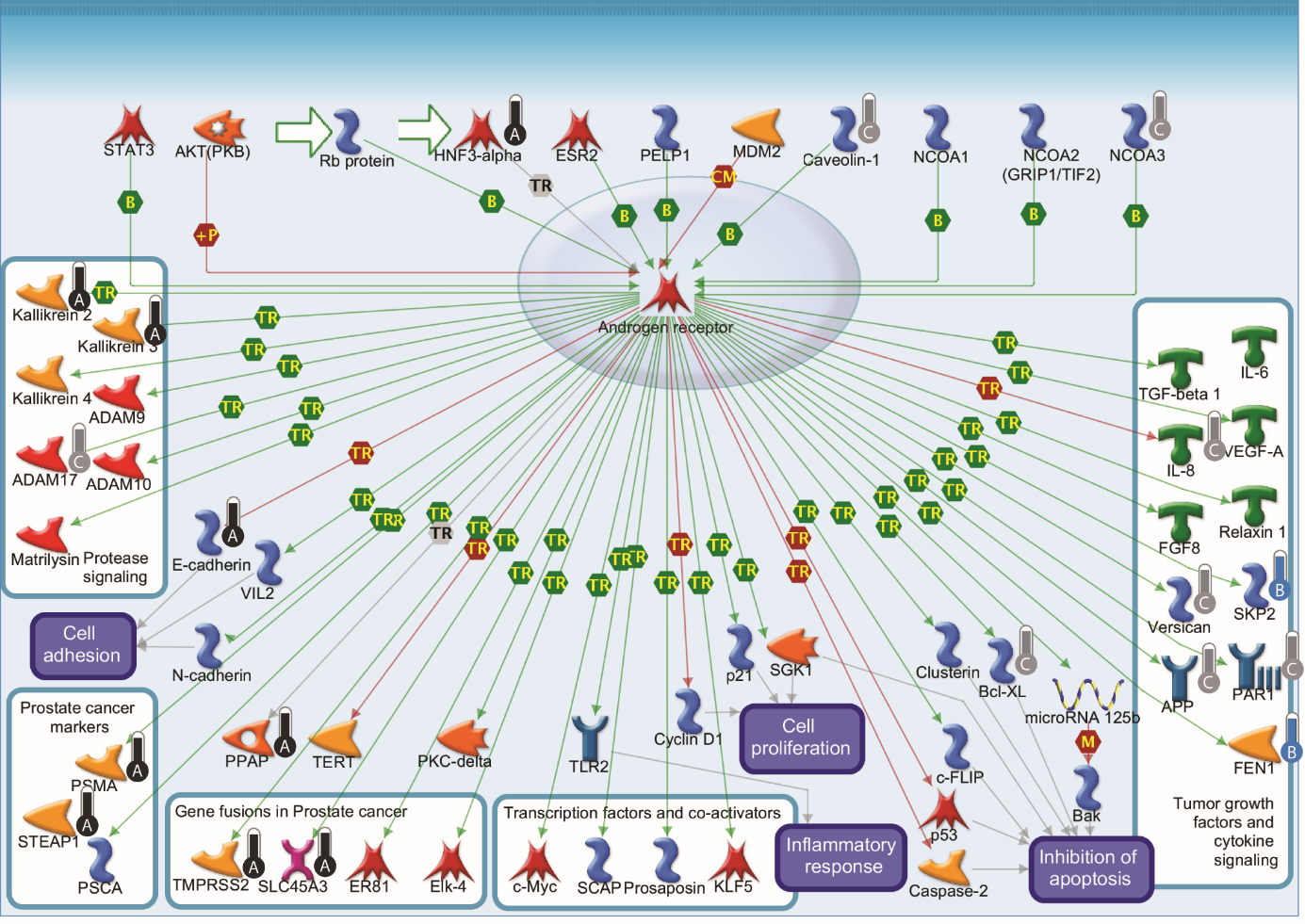


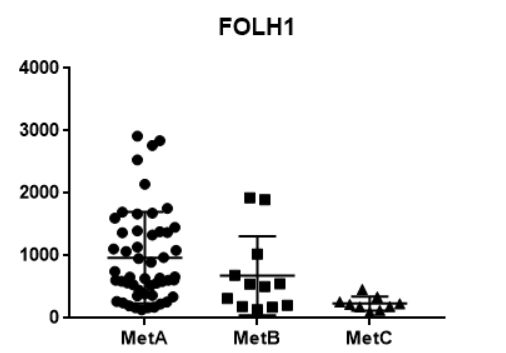

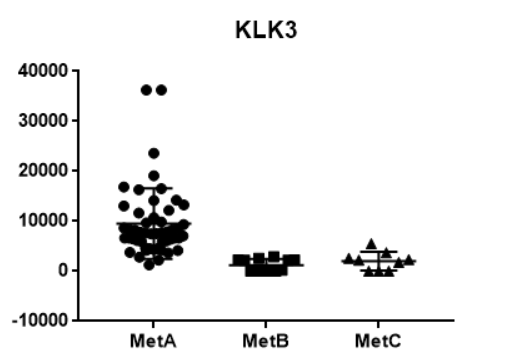

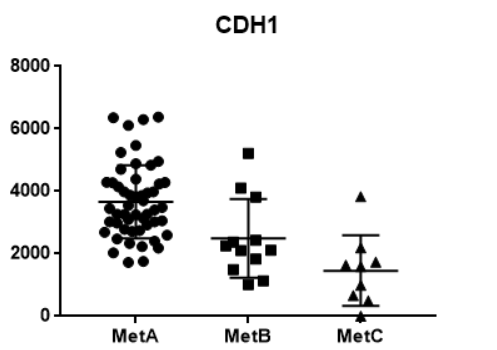

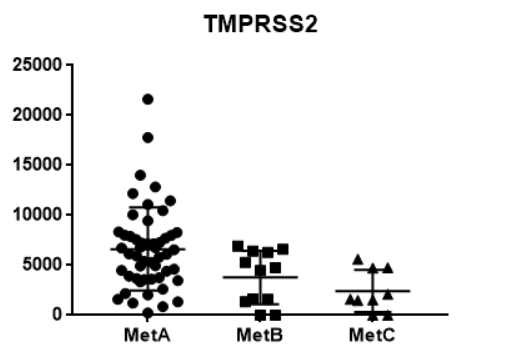


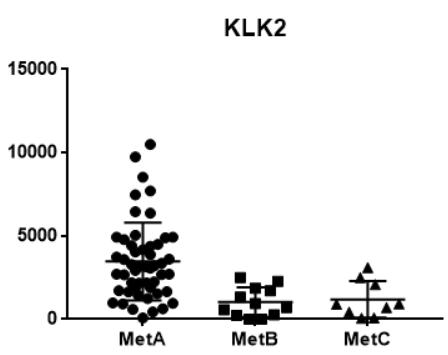

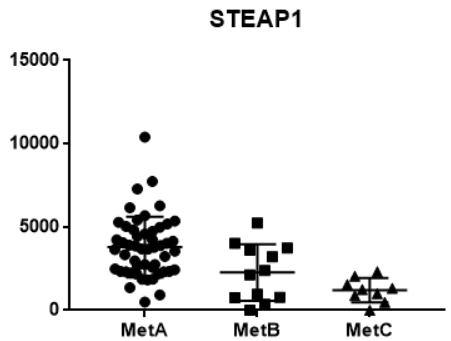

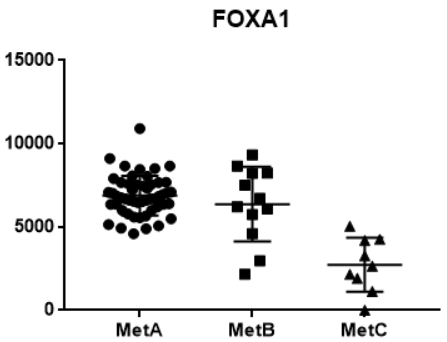

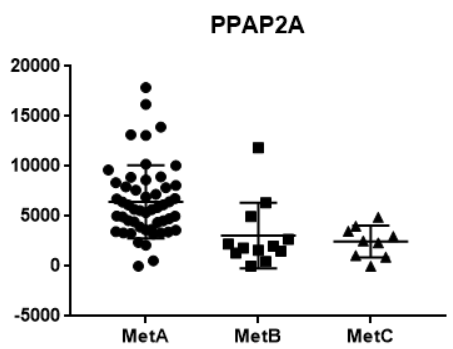


**Fig. S3.** Top functionally enriched pathway “Androgen receptor activation and downstream signaling in Prostate cancer” (Metacore software) in metastasis subtype MetA (see also Supporting Table S3), based on gene products of the pathway significantly enriched in Meta compared to MetB,C (black thermometers). Relative expression levels for selected genes are shown in box-plots. For symbol explanation, please see “the Metacore quick reference guide” (<https://portal.genego.com/help/MC_legend.pdf>)


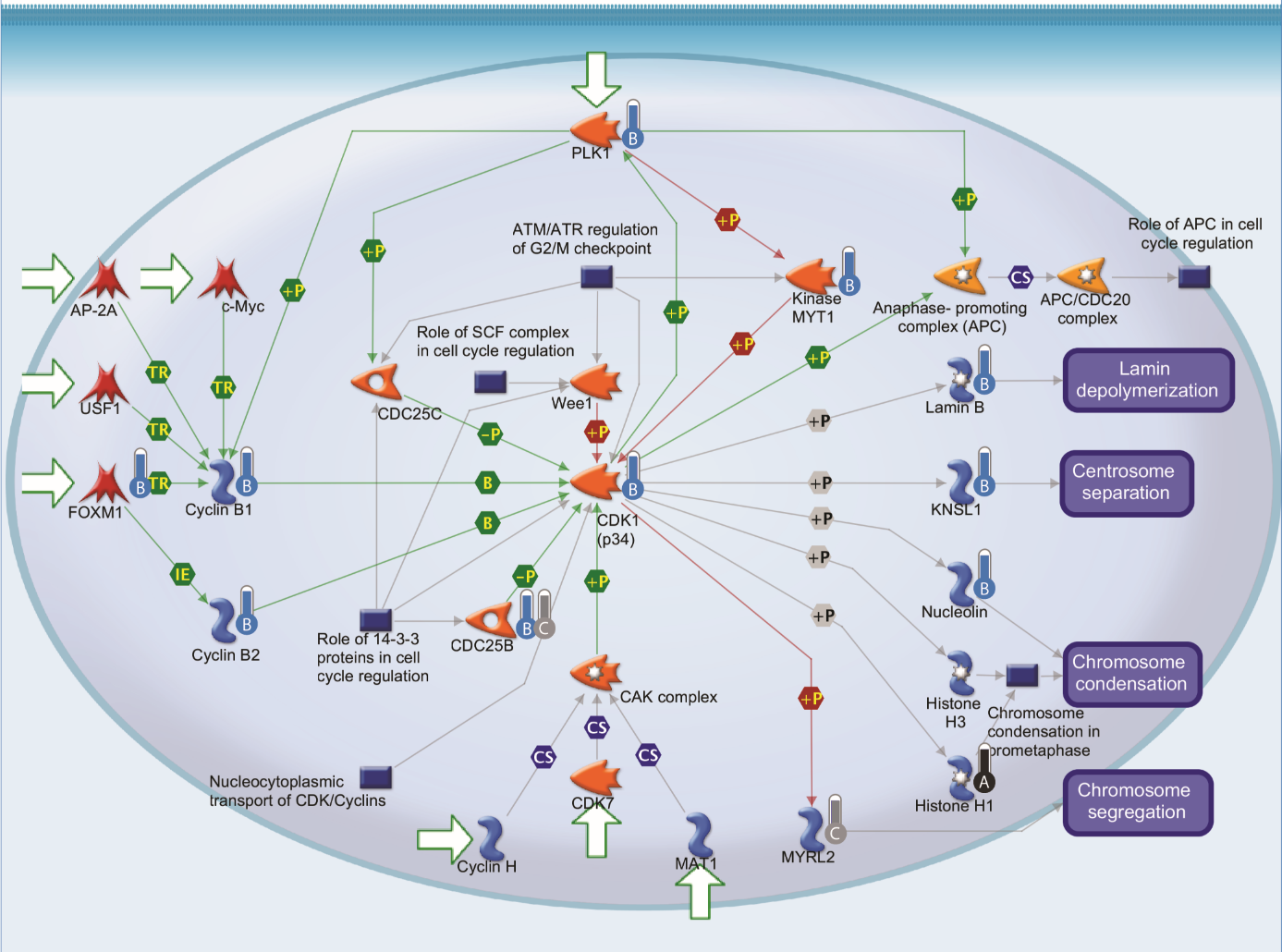


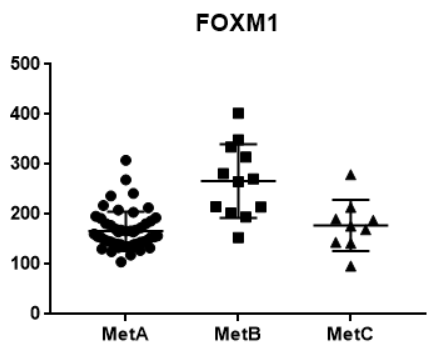

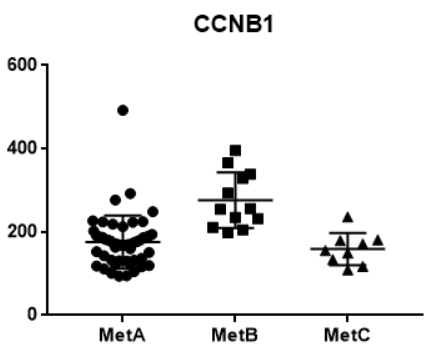

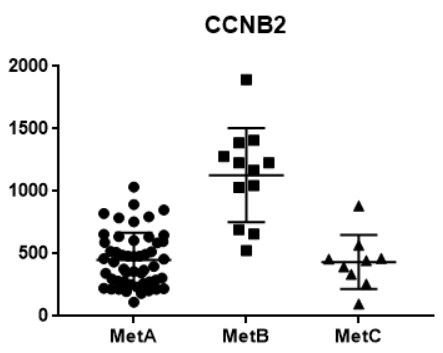

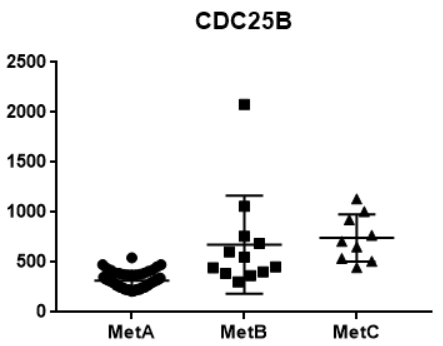


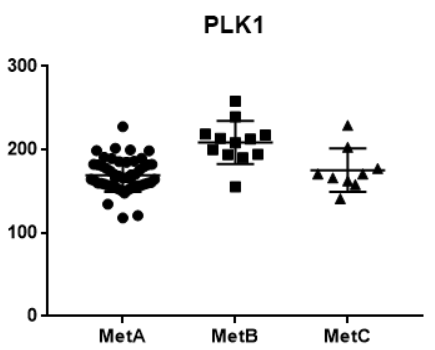

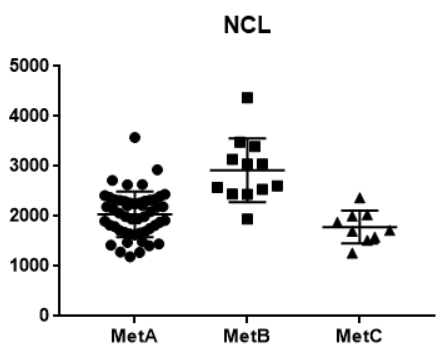

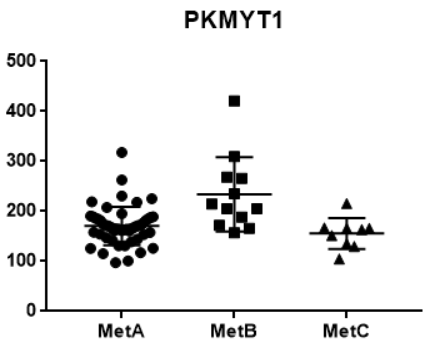

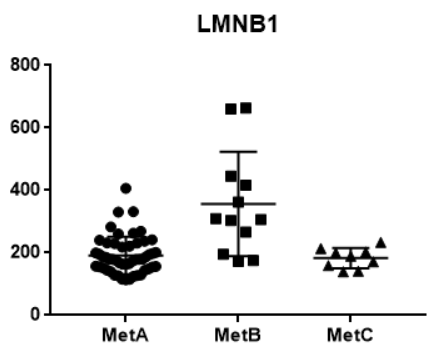


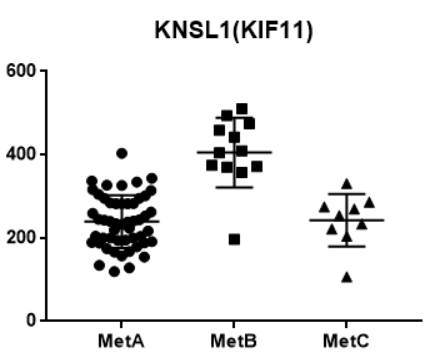

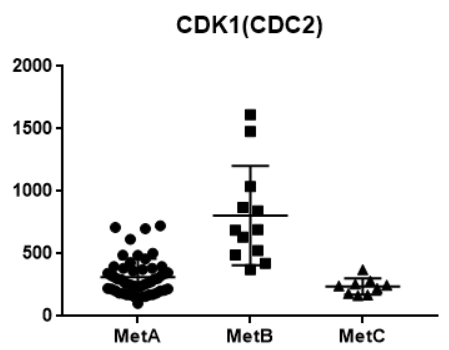


**Fig. S4.** Functionally enriched pathway map “Initiation of mitosis” (Metacore software) in metastasis subtype MetB (see also Supporting Table S3), based on gene products of the pathway significantly enriched in MetaB compared to MetA,C, (blue thermometers). Relative expression levels for selected genes are shown in box-plots. For symbol explanation, please see “the Metacore quick reference guide” (<https://portal.genego.com/help/MC_legend.pdf>)


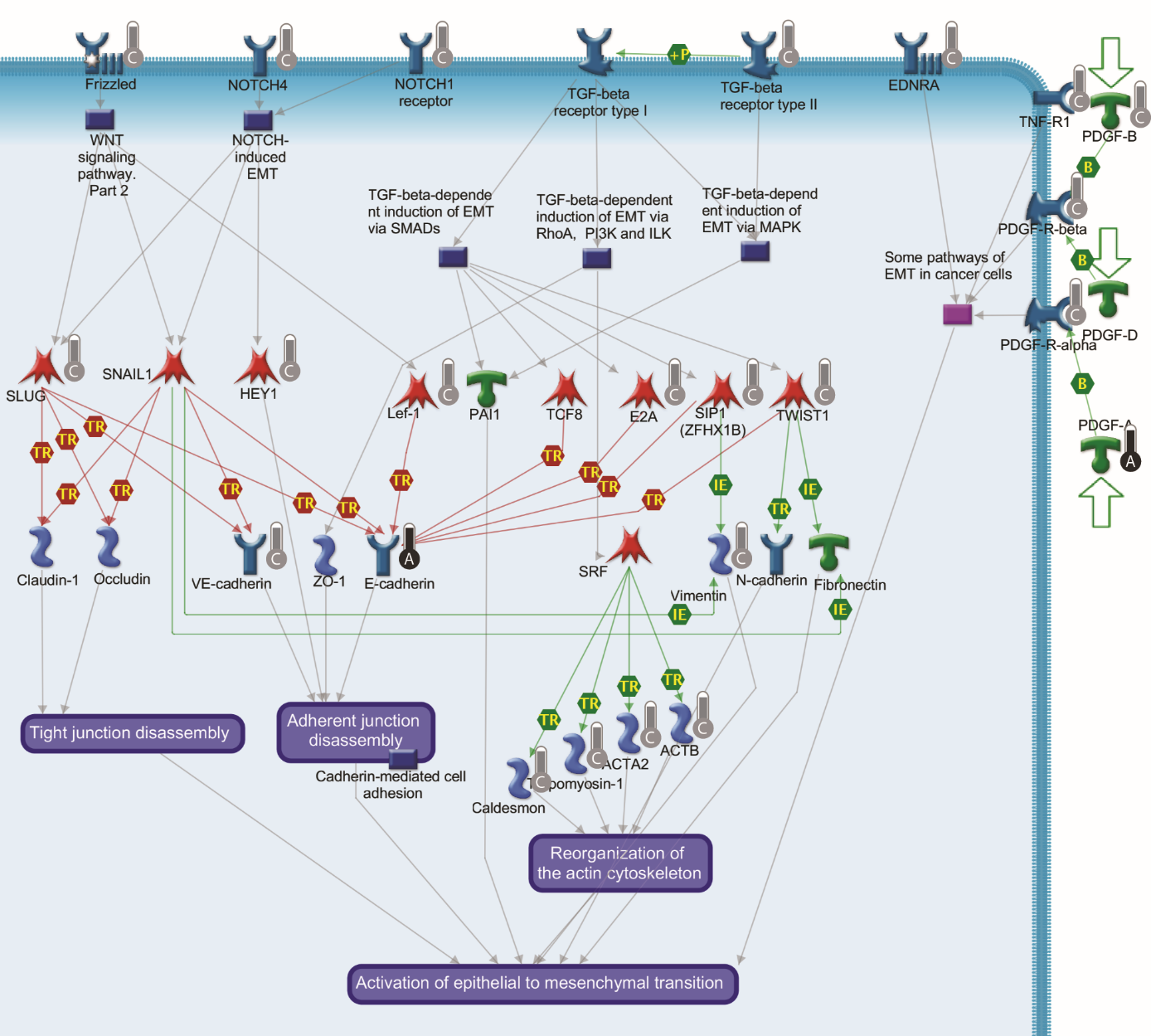


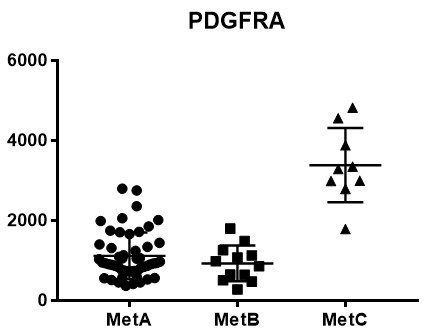

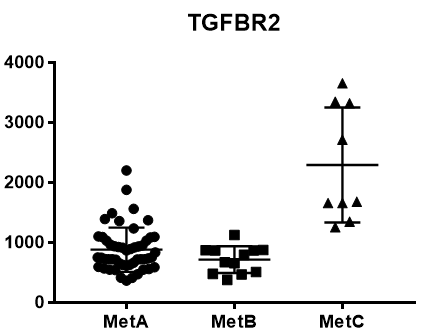

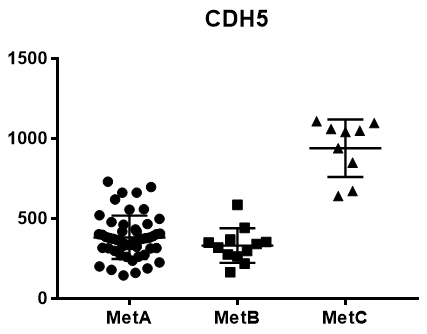

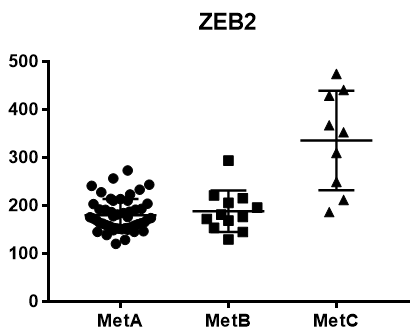


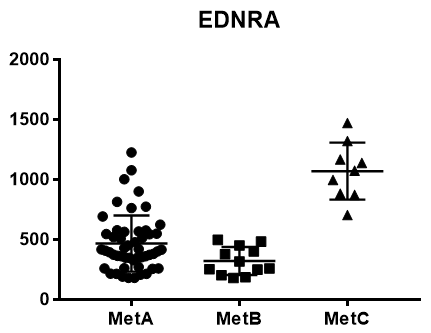

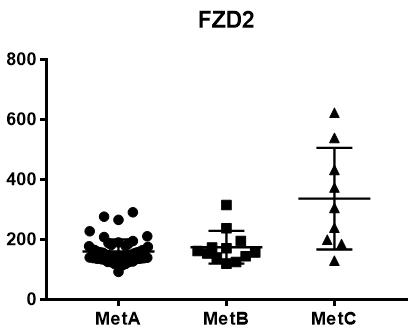

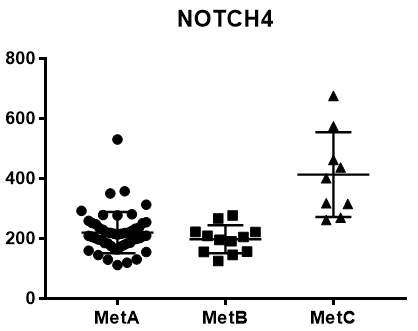


**Fig. S5.** The top two functionally enriched pathway map “Regulation of epithelial to mesenchymal transition” (Metacore software) in metastasis subtype MetC (see also Supporting Table S3), based on gene products of the pathway significantly enriched in MetC compared to MetA,B (gray thermometers). Relative expression levels for selected genes are shown in box-plots. For symbol explanation, please see “the Metacore quick reference guide” (<https://portal.genego.com/help/MC_legend.pdf> )


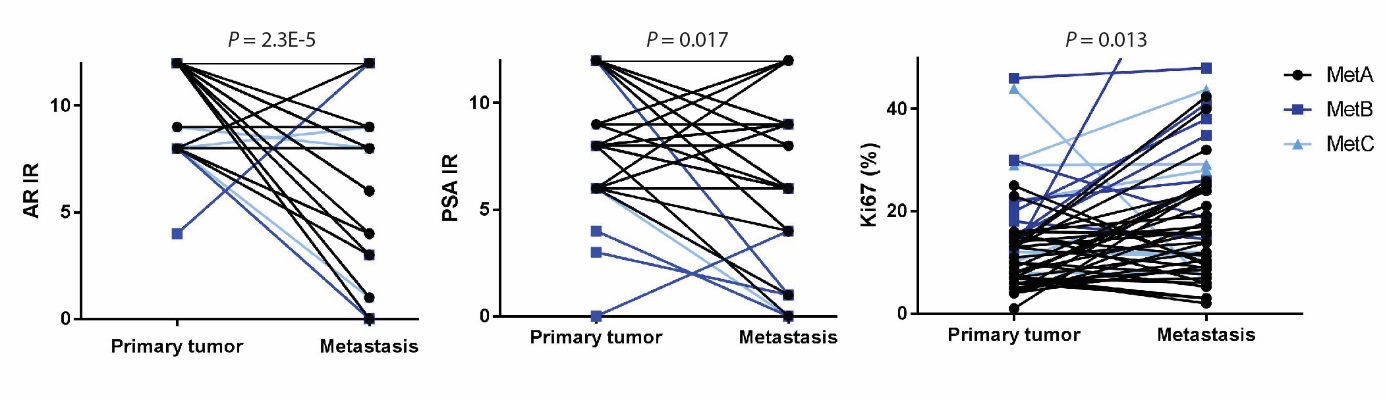


**Fig. S6.** Paired observations of androgen receptor (AR) (a), PSA (b) and Ki67 (c) immunoreactivity (IR) scores in bone metastases of subtypes A-C and in corresponding primary tumor biopsies. The AR and PSA IR were significantly reduced and the proliferation (fraction of Ki67 positive tumor cells) significantly increased in MetA metastases compared to their matched primary tumors.

**
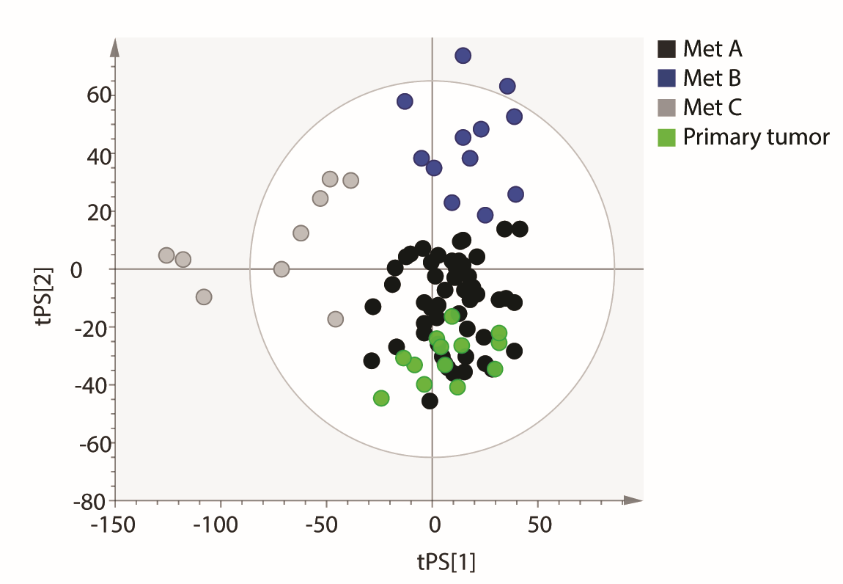
**

**Fig. S7.** Predictive score plot for the principal component analysis of 72 bone metastasis samples, based on whole genome expression analysis (Illumina bead chip array) (Fig. 1). Non-malignant prostate samples, analyzed in parallel with metastasis samples in (Hörnberg et al., 2011), cluster with MetA based on the gene expression pattern when predicted in the PCA model.
