# Supplementary material for: Gene expression profiles define molecular subtypes of prostate cancer bone metastases with different outcomes and morphology traceable back to the primary tumor
Source: Mol Oncol. 2019 Jun 27;13(8):1763–77. doi: 10.1002/1878-0261.12526 (PMC6670017; doi:10.1002/1878-0261.12526)
Supplement: Supplementary file 2 — Table S1. Principal component analysis model summary. [file MOL2-13-1763-s002.docx]

**Table S1**. Principal component analysis (PCA) model summary.

| **Principal Component** | **R2X** | **R2X(cum)** | **Eigenvalue** | **Q2** | **Q2(cum)** |
| --- | --- | --- | --- | --- | --- |
| 1 | 0.11 | 0.11 | 8.05 | 0.08 | 0.08 |
| 2 | 0.06 | 0.17 | 4.15 | 0.03 | 0.10 |
| 3 | 0.05 | 0.22 | 3.65 | 0.02 | 0.12 |
| 4 | 0.04 | 0.26 | 2.64 | 0.01 | 0.12 |
| 5 | 0.04 | 0.29 | 2.52 | 0.00 | 0.12 |
| 6 | 0.03 | 0.33 | 2.36 | 0.01 | 0.13 |
| 7 | 0.03 | 0.35 | 1.92 | 0.00 | 0.13 |
| 8 | 0.03 | 0.38 | 1.83 | 0.00 | 0.13 |
| 9 | 0.02 | 0.40 | 1.75 | 0.00 | 0.13 |

R2X - Fraction of Sum of Squares (SS) of the entire X explained by the current component.

R2X (cum) - Cumulative SS of the entire X explained by all extracted components.

Eigenvalue - Eigenvalue of the X matrix, R2X * min(K,N).

Q2 - The fraction of the total variation of X (PC)

Q2(cum) - The cumulative Q2 for all the x-variables (PC)
